# CFGSCDSA: Predicting circRNA-drug sensitivity associations based on collaborative feature learning and graph structure learning

**DOI:** 10.1371/journal.pcbi.1014072

**Published:** 2026-03-13

**Authors:** Xue Zhang, Quan Zou, Chunyu Wang, Mengting Niu

**Affiliations:** 1 Faculty of Computing, Harbin Institute of Technology, Harbin, China; 2 Yangtze Delta Region Institute (Quzhou), University of Electronic Science and Technology of China, Quzhou, China; 3 Institute of Fundamental and Frontier Sciences, University of Electronic Science and Technology of China, Chengdu, China; The University of Texas MD Anderson Cancer Center, UNITED STATES OF AMERICA

## Abstract

**Motivation:**

The expression of circular RNAs (circRNAs) has been shown to be strongly correlated with drug sensitivity in human cells. However, experimental validation using wet-lab techniques is costly and inefficient, leaving a substantial portion of circRNA–drug sensitivity associations undiscovered. Therefore, improving the prediction efficiency of circRNA and sensitivity associations remains critical.

**Methods:**

Here, we describe a method that integrates collaborative feature learning and graph structure learning to predict associations between circRNAs and drug sensitivity (CFGSCDSA). Specifically, collaborative learning integrated heterogeneous features from diverse data sources, thereby addressing the issue of data sparsity. Furthermore, graph structure learning with a confidence-guided pseudo-labeling strategy was employed to mitigate the detrimental effect of excessive negative samples. Results: Experimental evaluation revealed that CFGSCDSA attained superior performance compared to all competing models. Moreover, case studies provided further evidence of its capability to accurately predict both novel associations and new drug-related links.

## Author summary

Circular RNAs (circRNAs) play important roles in regulating drug sensitivity, but identifying their associations with specific drugs through biological experiments is costly and time-consuming. Computational methods therefore provide an efficient alternative for uncovering these potential relationships. In this study, we propose CFGSCDSA, a predictive framework that integrates collaborative feature learning with graph structure learning to improve the accuracy of circRNA–drug sensitivity association prediction. The collaborative feature-learning module effectively merges heterogeneous information from multiple biological sources, addressing data sparsity and enrich feature representations. A graph structure learning strategy guided by confidence-based pseudo-labeling strategy is employed to refine graph topology and mitigate the negative impact of false-negative samples. Extensive experiments demonstrate that CFGSCDSA outperforms existing state-of-the-art methods across multiple evaluation metrics. Furthermore, case studies verify its capability to uncover both known and novel circRNA–drug sensitivity associations, highlighting its potential to facilitate the discovery of therapeutic biomarkers.

## 1. Introduction

Circular RNAs (circRNAs) are a class of non-coding RNAs generated through back-splicing, resulting in covalently closed circular structures [1–3]. Research has shown that circRNAs modulate drug resistance and sensitivity by influencing diverse biological processes, including DNA repair mechanisms, immune evasion, and tumor-promoting inflammation [4–6]. Huang et al. demonstrated that CircESRP1 regulates the CDKN1A pathway, contributing to increased cisplatin sensitivity in small cell lung cancer [7]. Wang et al. showed that circ-METRN contributes to the radio-resistance of glioblastoma cells by regulating DNA damage repair via the miR-4709-3p/GRB14/PDGFRα cascade [8]. Qu et al. demonstrated that circCDYL2 enhances sensitivity in nasopharyngeal carcinoma by modulating the expression of RAD51 mRNA [9]. Additionally, circNOP14 sensitizes hepatocellular carcinoma cells to radiation through its interaction with Ku70 [10], and circCCAR1 contributes to anti-PD-1 immunotherapy resistance through modulation of the miR-127-5p feedback loop [11,12]. Collectively, this evidence highlights a robust relationship between circRNAs and drug sensitivity. Thus, investigating this relationship may facilitate the identification of potential therapeutic targets for cancer in clinical settings [13,14]. Accordingly, an urgent need exists to explore circRNAs–drug sensitivity associations(CDSAs) to better understand their roles in therapeutic response.

Traditional biological methods for identifying CDSAs are often expensive, labor-intensive, and inefficient [15–17]. Consequently, computational approaches have emerged as valuable tools for uncovering potential links between circRNAs and drug sensitivity [18–20]. GATECDA incorporates host gene sequences of circRNAs and drug structural information to compute similarity matrices, and then leverages a graph attention autoencoder to obtain low-dimensional embeddings for prediction [21]. Based on diverse data sources of circRNAs and drugs, MNGACDA establishes a multimodal network and then utilizes a graph attention autoencoder to extract node-level information [22]. MAGSDMF leverages multi-attention and graph learning methods to dynamically capture feature representations, followed by stacked deep matrix factorization for further feature refinement, and ultimately integrates the learned features using a multi-channel attention mechanism [23]. DPMGCDA first constructs homogenous and heterogeneous graphs for circRNAs and drugs, then extracts both global and fused features, and finally uses a path-masked graph autoencoder to predict their associations [24]. SNMGCDA extracts features using a sparse autoencoder, non-negative matrix factorization, and a multi-head graph attention network, and integrates them for final prediction [25]. HMFHCL constructs heterogeneous networks at both global and local levels for circRNAs and drugs, and enhances feature representation by performing matrix factorization and hypergraph convolution [26]. MiGNN2CDS introduces instance-level learning, designs a pseudo-metapath generator and a bidirectional translation embedding projection to learn metapath representations, and employs a multi-scale attention network for prediction [27]. Although these models have achieved certain progress in predicting CDSAs, several limitations remain. First, they fail to fully exploit data heterogeneity, leading to insufficient interaction learning. Second, CDSA datasets are relatively sparse, which may weaken the representation capability of the models.

**Fig 1:**
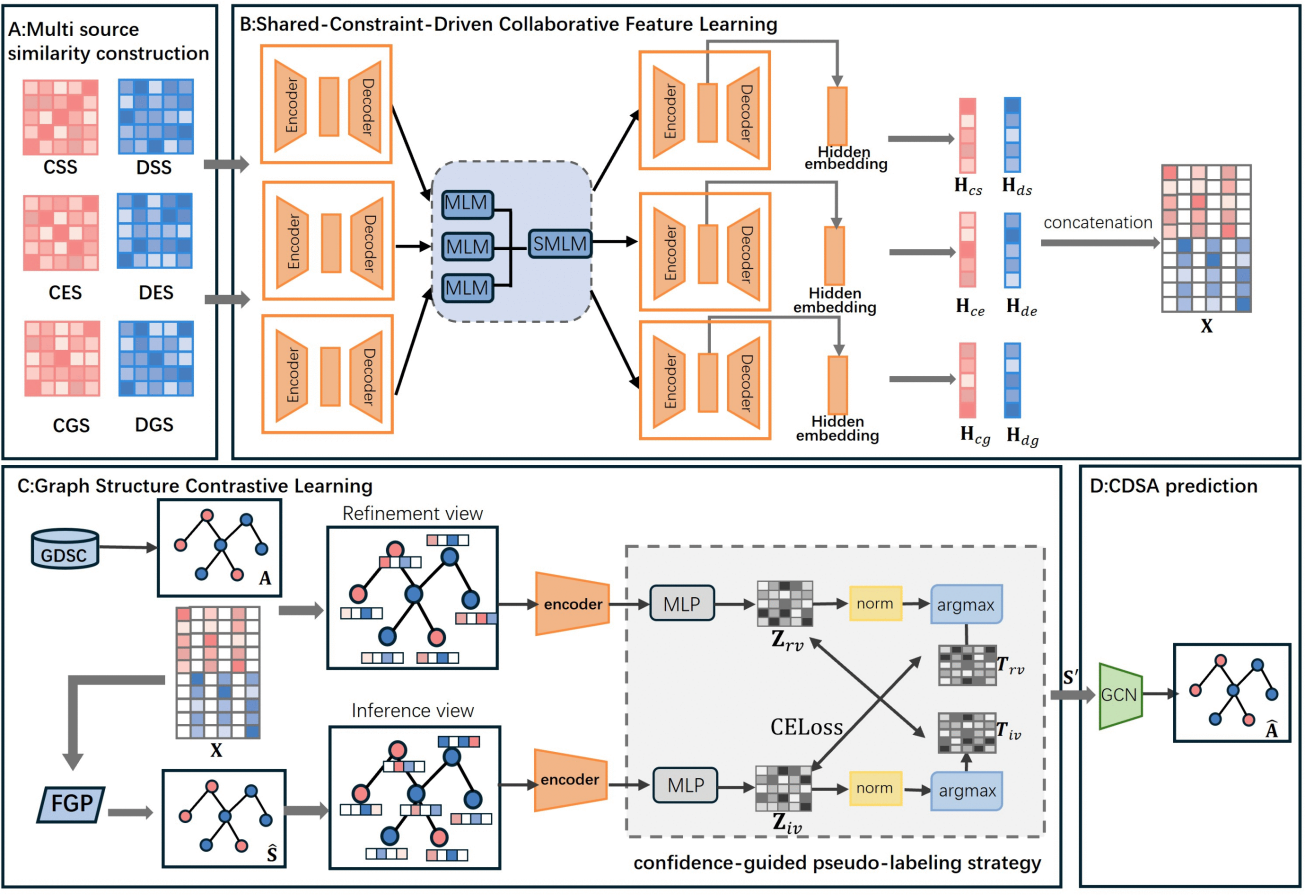
The overall workflow of CFGSCDSA. In step A, CFGSCDSA constructs multi-source similarity networks for circRNAs and drugs. In step B, the model employs collaborative feature learning strategy to comprehensively capture information from different data sources. In step C, graph structure learning with confidence-guided pseudo-labeling strategy is adopted to enhance the learning of topological features.

To effectively address these challenges, we propose CFGSCDSA, a novel framework that integrates collaborative feature learning with a confidence-guided pseudo-labeling based graph structure learning strategy. Unlike existing approaches, CFGSCDSA is specifically designed to jointly overcome the two major challenges of insufficient heterogeneous information utilization and dataset sparsity, thereby improving both interaction learning and topological representation robustness. The workflow of CFGSCDSA is illustrated in Fig 1. The CFGSCDSA model consists of the following three modules:

(1) Multi-source similarity construction, which incorporates multiple circRNA and drug similarity networks to provide enriched biological and topological information;
(2) Collaborative feature learning, which models and integrates differences among heterogeneous data sources, yielding more comprehensive representations and alleviating sparsity-induced overfitting;
(3) Confidence-guided pseudo-labeling based graph structure learning, which optimizes graph topology, enhances structural representation learning, and mitigates the influence of latent false-negative associations.

## 2. Materials and methods

### 2.1. Datasets

The dataset used in this study was sourced from reference [21], and obtained from the circRic database, where drug sensitivity data were derived from the Genomics of Drug Sensitivity in Cancer (GDSC) database [28]. The circRic database systematically profiled circRNA expression based on data from the Cancer Cell Line Encyclopedia (CCLE) and statistically analyzed the associations between circRNA expression and drug response. For each circRNA, the Wilcoxon test was employed to identify drugs whose sensitivities were significantly associated with circRNA expression. Notably, circRNAs in this dataset are labeled using their host gene names.

The original dataset comprised 80,076 circRNA–drug sensitivity associations involving 404 circRNAs and 250 drugs. Following significance filtering using a false discovery rate (FDR) threshold of 0.05, the final dataset used in this work consisted of 4,134 associations between 271 circRNAs and 218 drugs. These associations were encoded into an association matrix *Y*, where Y(i,j)=1 denotes the presence of an association between circRNA *i* and drug *j*, and Y(i,j)=0  denotes its absence.

These associations were encoded into an association matrix Y, where Y(i,j)=1 denotes an association and Y(i,j)=0 otherwise.

### 2.2. Data pre-processing and encoding

To obtain comprehensive and high-quality initial features, we computed six similarity matrices, including $CSS_{ij}$, $DSS_{ij}$, $CES_{ij}$, $DES_{ij}$, $CGS_{ij}$, and $DGS_{ij}$. Table 1 summarizes all similarity matrices used in this study, including their corresponding entity types, data sources, and computational strategies.

**Table 1:** Summary of similarity matrices used in CFGSCDSA.

Similarity matrix	Entity type	Description
$CSS_{ij}$	**circRNA–circRNA**	**Sequence-based similarity computed using Levenshtein distance between circRNA host gene sequences**
$DSS_{i,j}$	**drug–drug**	**Structural similarity calculated using the Tanimoto coefficient based on drug molecular fingerprints**
$CES_{ij}$	**circRNA–circRNA**	**Entropy-based similarity derived from shared circRNA association profiles**
$DES_{ij}$	**drug–drug**	**Entropy-based similarity derived from shared drug association profiles**
$CGS_{ij}$	**circRNA–circRNA**	**Gaussian interaction profile kernel similarity computed from circRNA–drug association matrix**
$DGS_{ij}$	**drug–drug**	**Gaussian interaction profile kernel similarity computed from circRNA–drug association matrix**

#### CircRNA sequence similarity.

We adopted the similarity calculation method for circRNAs proposed by Deng et al. [21]. Specifically, we first retrieved host gene information of circRNAs from the National Center for Biotechnology Information (NCBI) database [29] and then computed the similarity between host genes using the Levenshtein distance, which served as the similarity measure between circRNAs [30–32].

Let $s_{i}$ and $s_{j}$ denote the sequences of the host genes corresponding to circRNA *i* and circRNA *j*, respectively. The Levenshtein distance between them is computed via a dynamic programming matrix $LD\in R^{\left(|s_{i}|+\text{1})\times (|s_{j}|+\text{1}\right)}$, where each entry LD(p,q) represents the edit distance between the first *p* characters of $s_{i}$ and the first *q* characters of $s_{j}$. The recurrence relation is defined as:


$$\begin{array}{c} \begin{array}{c} \;LD(p,q)=\left\{ \;\;\;\;\;\;\begin{array}{ccc} @rlr & max(p,q)\text{ if }min(p,q)=\text{0}\\ & min\left\{\begin{array}{c} @cLD(p-\text{1},q)+\text{1},\\ LD(p,q-\text{1})+\text{1},\\ LD(p-\text{1},q-\text{1})+f(p,q) \end{array}\right\}\;otherwise\; & \end{array}\right.\;\; \end{array} \end{array}$$ (1)

where the mismatch indicator function f(p,q)is defined as:


$$\begin{array}{c} f(p,q)=\left\{ \begin{array}{c} @l\text{0},\;s_{i}[p]=s_{j}[q]\\ \text{1},\;s_{i}[p]\neq s_{j}[q]\; \end{array}\right.\; \end{array}$$ (2)

The complete edit distance between circRNA *i* and *j* is obtained at:


$$\begin{array}{c} LD\left(s_{i},s_{j}\right)=LD\left(\left|s_{i}\right|,\left|s_{j}\right|\right) \end{array}$$ (3)

To normalize this distance and transform it into a similarity score, the circRNA sequence similarity matrix $CSS_{ij}\;$ is defined as:


$$\begin{array}{c} CSS_{ij}=\text{1}-\frac{LD\left(s_{i},s_{j}\right)}{max\left(\left|s_{i}\right|,\left|s_{j}\right|\right)} \end{array}$$ (4)

#### Drug structure similarity.

Analyzing the chemical structure of drugs enables the evaluation of their functional similarity. Specifically, SMILES structural data were obtained from the PubChem database [33], topological fingerprints were generated using RDKit, and structural similarity $\text{DS}{\text{S}}_{i,j}$ was computed using the Tanimoto coefficient.


$$\begin{array}{c} \text{DS}{\text{S}}_{i,j}=\frac{d_{i}\cdot d_{j}}{{\left|d_{i}\right|}^{\text{2}}+{\left|d_{j}\right|}^{\text{2}}-d_{i}\cdot d_{j}} \end{array}$$ (5)

Here, $d_{i}$ and $d_{j}$ correspond to the topological fingerprint vectors of drugs *i* and *j*.

#### Entropy similarity of circRNA and drug.

The concept of information entropy was originally introduced by Shannon [34,35]. In this study, we employ entropy-based methods to independently compute the similarity between circRNAs and drugs. Taking circRNAs as an example, where $S_{i}=d_{i}^{\left(\text{1}\right)},d_{i}^{\left(\text{2}\right)},\ldots ,d_{i}^{\left(n_{i}\right)}$ denotes the drug set associated with circRNA *i*. For each drug $d_{i}^{\left(k\right)}\in S_{i}$, the occurrence probability is defined as:


$$\begin{array}{c} p_{i}^{\left(k\right)}=\frac{n\left(d_{i}^{\left(k\right)}\right)}{N} \end{array}$$ (6)

Where *N* represent the total number of known circRNA–drug associations, $n\left(d_{i}^{\left(k\right)}\right)$ denotes the number of circRNAs known to be associated with drug $d_{i}^{\left(k\right)}$ The information entropy of circRNA *i* is then calculated as:


$$\begin{array}{c} H\left(S_{i}\right)=-\sum_{k=\text{1}}^{n_{i}}\;p_{i}^{\left(k\right)}{\text{log}}_{\text{2}}p_{i}^{\left(k\right)} \end{array}$$ (7)

The entropy-based similarity between circRNA *i* and circRNA *j* is defined as:


$$\begin{array}{c} CES_{ij}=\frac{\text{2}\times H\left(S_{i}\cap S_{j}\right)}{H\left(S_{i}\right)+H\left(S_{j}\right)} \end{array}$$ (8)

$S_{i}\cap S_{j}$ refers to the set of drugs that exhibit associations with both circRNA *i* and circRNA *j*.

The entropy similarity $DES_{ij}$ is then calculated using the same formula:

#### Gaussian kernel function similarity of circRNAs and drugs.

The Gaussian interaction profile (GIP) kernel similarity was incorporated to obtain more comprehensive features [36]. The GIP similarity matrix $CGS_{ij}$ for circRNAs was determined as follows:


$$\begin{array}{c} CGS_{ij}=exp(-\theta_{c}||Y(c(i))-Y(c(j))|{|}^{\text{2}}) \end{array}$$ (9)

$$\begin{array}{c} \theta_{c}=\text{1}/\left(\frac{\text{1}}{n}\sum_{i=\text{1}}^{n}\;∥Y\left(c(i)\right){∥}^{\text{2}}\right) \end{array}$$ (10)

Here, $\theta_{\text{c}}$ denotes the bandwidth parameter for circRNAs, and n represents the total number of circRNAs. Y(c(i)) refers to the column in the association matrix corresponding to circRNA *i*.

The GIP similarity matrix $DGS_{ij}$ for drugs was determined as follows:


$$\begin{array}{c} DGS_{ij}=\text{exp}(-\theta_{d}||Y(d(i))-Y(d(j))|{|}^{\text{2}}) \end{array}$$ (11)

$$\begin{array}{c} \theta_{d}=\text{1}/\left(\frac{\text{1}}{m}\sum_{i=\text{1}}^{m}\;∥Y\left(d(i)\right){∥}^{\text{2}}\right) \end{array}$$ (12)

Here, $\theta_{d}$ denotes the bandwidth parameter for drugs, and *m* represents the total number of drugs. Y(d(i)) refers to the row in the association matrix corresponding to drug *i*.

### 2.3. Network structure

#### 2.3.1. Shared-constraint-driven collaborative feature learning.

To mitigate the weak interactions between heterogeneous sources, we adopted a shared-constraint-driven collaborative feature learning approach.

**Feature Encoding:** The similarity network was used as the input feature to the autoencoder. Specifically, the circRNA similarity network $G_{\text{c}}=G_{\text{c}}^{\left(\text{1}\right)},G_{\text{c}}^{\left(\text{2}\right)},\ldots ,G_{\text{c}}^{\left(\text{k}\right)}$ was encoded using a Graph Convolutional Network(GCN) to generate node representations,


$$\begin{array}{c} \begin{array}{c} H^{\left(\text{1}\right)}=g_{\phi }^{\left(\text{1}\right)}\left(G_{\text{c}}^{\left(\text{1}\right)}\right)\\ \ldots \\ H(k)=g_{\phi }^{\left(k\right)}\left(G_{\text{c}}^{\left(\text{k}\right)}\right) \end{array} \end{array}$$ (13)

where H(k) denotes the node features of $G_{\text{c}}^{\left(\text{k}\right)}$, and $g_{\phi }^{\left(k\right)}$ denotes the encoder.

**Shared Constraint:** We determined shared constraints based on the similarity between nodes. In semi-supervised clustering, nodes with high similarity are referred to as “must-link” pairs. Specifically, Pearson correlation coefficients (PCC) were employed to compute pairwise node similarities $\text{Sim}\left(c_{i}^{\left(k\right)},c_{j}^{\left(k\right)}\right)$:


$$\begin{array}{c} \text{Sim}\left(c_{i}^{\left(k\right)},c_{j}^{\left(k\right)}\right)=\text{PCC}\left({\text{h}}_{i}^{\left(k\right)},{\text{h}}_{j}^{\left(k\right)}\right) \end{array}$$ (14)

$$\begin{array}{c} \text{PCC}\left({\text{h}}_{i}^{\left(k\right)},{\text{h}}_{j}^{\left(k\right)}\right)=\frac{\sum_{c=\text{1}}^{N_{d}}\;\left({\text{h}}_{ic}-{\overset{―}{\text{h}}}_{i}\right)\left({\text{h}}_{jc}-{\overset{―}{\text{h}}}_{j}\right)}{\sqrt{\sum_{c=\text{1}}^{N_{d}}\;({\text{h}}_{ic}-{\overset{―}{\text{h}}}_{i}{)}^{\text{2}}}\cdot \sqrt{\sum_{c=\text{1}}^{N_{d}}\;({\text{h}}_{jc}-{\overset{―}{\text{h}}}_{j}{)}^{\text{2}}}} \end{array}$$ (15)

In this context, $h_{k}^{i}$ and $h_{k}^{j}$ refer to the feature representations of $c_{i}$ and $c_{j}$ within $H_{k}$, while $N_{d}$ indicates the dimension of the feature space.

After obtaining the PCC values from all circRNA networks, a subset of entries is selected. The corresponding positions are set to 1 while the rest are set to 0, resulting in the “must-link” constraint matrix MLM.


$$\begin{array}{c} \text{MLM}\left(c_{i}^{\left(k\right)},c_{j}^{\left(k\right)}\right)=\left\{ \begin{array}{c} @l\text{1}\text{ if Sim}\left(c_{i}^{\left(k\right)},c_{j}^{\left(k\right)}\right)\in \text{ML}\\ \text{0}\text{ otherwise} \end{array}\right.\; \end{array}$$ (16)

All similarity attributes are subsequently utilized to jointly extract circRNA feature representations, and the shared of all “must-link” constraint matrix SMLM is defined as the shared constraint.


$$\begin{array}{c} \text{SMLM}=\cap_{i=\text{1}}^{k}{\text{MLM}}_{i} \end{array}$$ (17)

Specifically, ∩ denotes the operation that computes the intersection between sets.

This shared constraint, together with the low-dimensional features obtained from the first autoencoder, is then fed into the subsequent autoencoder.


$$\begin{array}{c} \begin{array}{c} {\text{Y}}_{m}^{(\text{1}{)}^{\prime }}=f_{\theta }^{\left(\text{1}\right)}\left({\text{H}}^{\left(\text{1}\right)}\right)\\ \ldots \\ {\text{Y}}_{m}^{(k{)}^{\prime }}=f_{\theta }^{\left(k\right)}\left({\text{H}}^{\left(k\right)}\right) \end{array} \end{array}$$ (18)

Here, $f_{\theta }^{\left(k\right)}$ represents the decoder.

**Loss Function Construction:** We first constructed a loss function $L_{reco}$ based on the original and the reconstructed features obtained through the encoder-decoder framework.


$$\begin{array}{c} L_{reco}=\sum_{n=\text{1}}^{k}\;\frac{∥{\text{Y}}_{m}^{\left(n\right)}-{\text{Y}}_{m}^{(n{)}^{\prime }}{∥}^{\text{2}}}{k} \end{array}$$ (19)

Here, *k* denotes the number of circRNA nodes.

Subsequently, we introduced a shared constraint loss to enforce similarity between feature representations. Specifically, for nodes and that form a *must-link* pair, their embeddings are required to be proximal in the feature space. The corresponding loss function $L_{mc}$ is defined as:


$$L_{mc}=\frac{\text{1}}{\left|S\right|}\left(\sum_{<h_{i},h_{j}>\in \text{SMLM}}\;∥h_{i}-h_{j}{∥}^{\text{2}}\right)$$ (20)

where $h_{i}$ and $h_{j}$ correspond to the features in the vector space ${\text{Y}}_{m}^{(k{)}^{\prime }}$, while *S* indicates the number of shared constraint pairs.

Therefore, the overall loss function of the collaborative feature extraction module $L_{CFE}$ is defined as follows:


$$\begin{array}{c} L_{CFE}=L_{reco}+L_{mc} \end{array}$$ (21)

After applying collaborative feature learning, similarity features of circRNAs and drugs were obtained and then aggregated to form the feature matrix *X*. To integrate heterogeneous circRNA and drug features, we construct a block-structured feature matrix *X*. Zero matrices are inserted between circRNA and drug feature blocks to prevent unintended cross-type interactions. This block structure preserves the independence of the two biological entities while enabling unified feature representation learning.


$$\begin{array}{c} \begin{array}{c} \text{X}=[\begin{array}{cccccc} {\text{H}}_{cs} & \text{0} & {\text{H}}_{ce} & \text{0} & {\text{H}}_{cg} & \text{0}\\ \text{0} & {\text{H}}_{ds} & \text{0} & {\text{H}}_{de} & \text{0} & {\text{H}}_{dg} \end{array}] \end{array} \end{array}$$ (22)

#### 2.3.2. Graph structure learning.

To reduce the influence of latent false-negative samples in the CDSA dataset, we incorporate a confidence-guided pseudo-labeling mechanism into graph structure learning. This design reduces the impact of low-confidence negative samples, preventing potentially false-negative pairs from dominating the graph structure learning process and yielding a more reliable circRNA–drug interaction topology.

**Graph learner:** Graph learners are capable of modeling graph structures. We adopted the FGP learner to model the adjacency matrix S as follows:


$$\begin{array}{c} \text{S}=\sigma (\text{A}) \end{array}$$ (23)

$$\begin{array}{c} \begin{array}{c} \text{A}=[\begin{array}{cc} \text{0} & \text{Y}\\ Y^{T} & \text{0} \end{array}] \end{array} \end{array}$$ (24)

where σ is defined as the nonlinear activation function.

The adjacency matrix S is normalized and symmetrized to ensure consistency, resulting in $\hat{\text{S}}$.


$$\begin{array}{c} {\hat{S}}_{\text{sym}}=\frac{\sigma (\text{S})+\sigma (\text{S}{)}^{\text{T}}}{\text{2}} \end{array}$$ (25)

$$\begin{array}{c} \hat{\text{S}}={\hat{S}}_{norm}={\hat{D}}^{-\frac{\text{1}}{\text{2}}}{\hat{S}}_{sym}{\hat{D}}^{-\frac{\text{1}}{\text{2}}} \end{array}$$ (26)

Here, D denotes the degree matrix of *S*, and σ represents the activation function.

**View Construction:** To enhance feature representation, we adopted an inference and an optimization view as dual perspectives in the learning process. The refined view $G_{rv}$ was constructed based on the original topological structure and the features extracted by the collaborative feature learning module.


$$\begin{array}{c} G_{rv}=(\text{A},\text{X}) \end{array}$$ (27)

The inference view $G_{iv}\;$ is composed of an adjacency matrix $\hat{\text{S}}$ generated by the graph learner and feature representations derived from the collaborative feature learning module.


$$\begin{array}{c} G_{iv}=\left(\hat{\text{S}},\text{X}\right) \end{array}$$ (28)

**Data Augmentation:** When two views exhibit high similarity, the learning of discriminative features becomes limited [37,38]. To mitigate this, we incorporated edge dropout and feature masking across the two views, thereby inducing structural variation and facilitating the extraction of richer semantic features.

Different feature masking probabilities are applied to the two views, where p(mr) is used for the refinement view and p(mi) for the inference view.


$$\begin{array}{c} {\overset{―}{X}}_{mr}={\left[x_{\text{1}}⨀\beta^{\left(r\right)},x_{\text{2}}⨀\beta^{\left(r\right)},\ldots ,x_{n}⨀\beta^{\left(r\right)}\right]}^{\text{T}} \end{array}$$ (29)

$$\begin{array}{c} {\overset{―}{X}}_{mi}={\left[x_{\text{1}}⨀\beta^{\left(i\right)},x_{\text{2}}⨀\beta^{\left(i\right)},\ldots ,x_{n}⨀\beta^{\left(i\right)}\right]}^{\text{T}} \end{array}$$ (30)

${\overset{―}{X}}_{mr}$ and ${\overset{―}{X}}_{mi}$ denote the feature matrix of the inference view after feature masking, where $x_{i}$ represents the transpose of the *i*-th row feature of *X*, and $\beta^{\left(r\right)}$ and $\beta^{\left(i\right)}$ are the masking vectors generated according to a Bernoulli distribution with the masking probability p(mr) and p(mi).

Feature masking resulted in significantly different graph structures of the two views; thus, the same edge dropout probability was applied to both views. The adjacency matrices of the refined and inference views after edge dropout are defined as follows:


$$\begin{array}{c} {\overset{―}{A}}_{rv}=A⨀\beta^{\left(e\right)},{\overset{―}{S}}_{iv}=\hat{S}⨀\beta^{\left(e\right)} \end{array}$$ (31)

$\beta^{\left(e\right)}$ is the dropping vector generated from the Bernoulli distribution with the dropping probability p(e). ${\overset{―}{A}}_{rv}$ and ${\overset{―}{S}}_{iv}$ represent the adjacency matrices of the defined view and inference view after edge dropout, respectively. The augmented views are thus obtained as follows:


$$\begin{array}{c} {\overset{―}{G}}_{rv}=\left({\overset{―}{A}}_{rv},{\overset{―}{X}}_{mr}\right),{\overset{―}{G}}_{iv}=\left({\overset{―}{S}}_{iv},{\overset{―}{X}}_{mi}\right) \end{array}$$ (32)

For both the refined view and the inference view, representations $H_{rv}$ and $H_{iv}$ are obtained from the augmented views via an encoder, and then mapped to feature vectors $Z_{rv}$ and $Z_{iv}$ through Multilayer Perceptron (MLP). The process is defined as follows:


$$\begin{array}{c} H_{rv}=f_{rv}\left({\overset{―}{G}}_{rv}\right),Z_{rv}=g_{rv}\left(H_{rv}\right) \end{array}$$ (33)

$$\begin{array}{c} H_{iv}=f_{iv}({\overset{―}{G}}_{iv}),Z_{iv}=g_{iv}(H_{iv}) \end{array}$$ (34)

Here, *f* and *g* denote the parameters of the GCN encoder and the MLP layer, respectively.

**Confidence-guided Pseudo-labeling:** Inspired by the minimum-entropy principle in self-supervised learning [39], we design a confidence-guided pseudo-labeling strategy to provide reliable supervision for cross-view representation learning. It consists of three key steps. The first step involves normalizing the feature representations:


$$\begin{array}{c} {\text{N}}_{rv}=norm\left({\text{Z}}_{rv}\right),{\text{N}}_{\text{iv}}=norm\left({\text{Z}}_{\text{iv}}\right) \end{array}$$ (35)

Then, compute the index of the dimension with the maximum value in the normalized feature vector.


$$\begin{array}{c} T_{rv}=argmax\left(N_{rv}\right),T_{iv}=argmax\left(N_{iv}\right) \end{array}$$ (36)

Finally, the loss function $L_{cpl}\;$ is based on cross-entropy loss, as follows:


$$\begin{array}{c} L_{cpl}=\frac{\text{1}}{\text{2}}\left(\text{CELoss}\left(Z_{rv},T_{rv}\right)+\text{CELoss}\left(Z_{iv},T_{iv}\right)\right) \end{array}$$ (37)

where CELoss means cross-entropy loss.

#### 2.3.3. CDSA prediction.

The optimal graph structure ${\text{S}}^{\prime }$ was obtained through the semi-supervised learning module, after which a GCN was employed to predict CDSA associations, resulting in the prediction result matrix $\hat{\text{A}}$.


$$\begin{array}{c} \hat{\text{A}}=GCN\left(\text{X},{\text{S}}^{\prime }\right)\; \end{array}$$ (38)

The loss function $L_{pre}$ employs mean squared error (MSE) loss to minimize the discrepancy between the predicted values and the ground truth.


$$L_{pre}=\frac{\text{1}}{N}\sum_{i=\text{1}}^{N}\;({\hat{y}}_{i}-y_{i}{)}^{\text{2}}$$ (39)

The predicted value of the i-th sample in set $\hat{\text{A}}$ is denoted by ${\hat{y}}_{i}$, while the true value of the i-th sample in set A is represented by $y_{i}$.

## 3. Results and discussion

### 3.1. Performance evaluation

**Fig 2:**
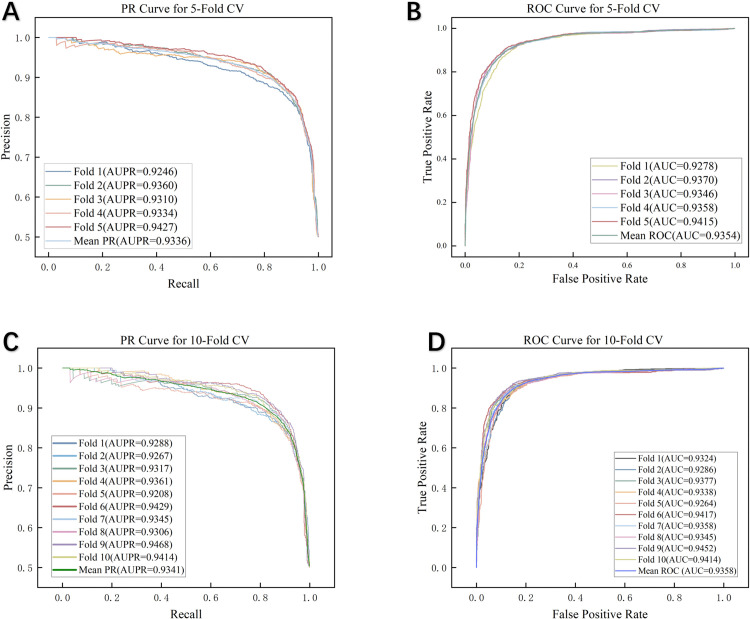
The results of PR curves and ROC curves of CFGSCDSA under 5-cv and 10-cv.

In the experiments, the ratio of positive to negative samples was set to 1:1. We used 5-fold and 10-fold cross-validation (CV) to evaluate the model’s predictive ability [40,41]. Under 5-fold CV, CFGSCDSA achieved an area under the curve (AUC) of 0.9354 and an area under the precision–recall curve (AUPR) of 0.9336, as illustrated in Fig 2. For 10-fold CV, the model attained an AUC of 0.9358 and an AUPR of 0.9341.

### 3.2. Comparison with other methods

To assess the effectiveness of CFGSCDSA, we compared it against seven representative state-of-the-art CDSA prediction methods: MHCDA [42], DHANMKF [43], DGATCCDA [44], MNGACDA [22], MNCLCDA [45], GATECDA [21], and MHGTCDA [46]. MHCDA leverages GCN and hypergraph convolution to capture local and global information. DHANMKF applies intra and inter-type attention mechanisms. DGATCCDA uses a DeepWalk-enhanced graph attention network for multimodal node embedding. MNGACDA employs a graph autoencoder with node-level attention. MNCLCDA integrates hybrid neighborhood GCN with graph contrastive learning. GATECDA extracts low-dimensional features using a graph attention autoencoder, and MHGTCDA combines an adaptive encoder with a multi-layer heterogeneous graph transformer for association prediction. For a fair comparison, all competing methods were implemented using the parameter settings recommended in their original publications.

**Fig 3:**
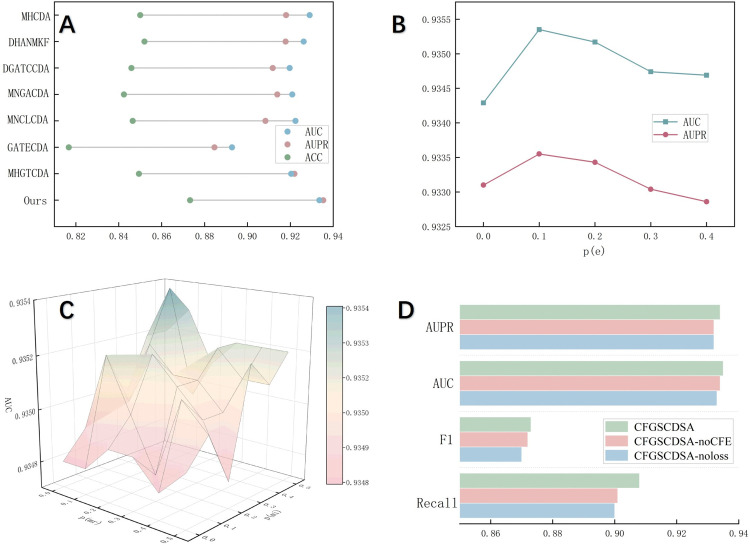
(A) Performance comparison among different methods. **(B)** Parameter analysis for edge dropout. **(C)** Parameter analysis for masking probability. **(D)** Performance of ablation study.

We describe three evaluation metrics—AUC, AUPR, and accuracy. The results of 5-CV for the eight methods are shown in Fig 3A. CFGSCDSA outperformed GATECDA, MNCLCDA, and MNGACDA in terms of AUC by 5.08%, 2.7%, and 2.34%, respectively, and exceeds the second-best model by 1.35%, indicating that CFGSCDSA consistently outperformed existing CDSA prediction approaches in all metrics, thereby validating the effectiveness of the proposed model. While existing approaches employ different strategies to extract circRNA and drug features, they insufficiently consider the correlations within homogeneous similarity networks. Moreover, the proposed model integrates the confidence-guided pseudo-labeling strategy to reduce the adverse effects of unreliable negative samples and improve feature robustness. These findings demonstrate the effectiveness of CFGSCDSA for CDSA prediction, underscoring its capability to enhance feature representation via collaborative learning and self-supervised mechanisms.

### 3.3. Parameter sensitivity analysis

Hyperparameter settings significantly affect model performance. We primarily analyzed the effects of masking probability p(mr) and p(mi), as well as edge dropout probability p(e) on the outcome.

We first evaluated edge dropout probability, as removing edges enhances performance during graph structure learning. The results under varying edge dropout probability settings are depicted in Fig 3B. Optimal performance was obtained at p(e) = 0.1, whereas higher values result in progressively decreased performance.

Further, we applied distinct masking probabilities p(mr) and p(mi), respectively. Fig 3C presents the AUC values under all combinations of masking probabilities. The highest AUC was achieved when p(mr)=0.1 and p(mi)=0.4, whereas other combinations resulted in varying degrees of performance degradation.

### 3.4. Ablation study

To assess the contribution of our modifications, we constructed two ablated variants: (1) CFGSCDSA-noCFE, in which the collaborative feature learning module was removed and the input was formed by concatenating similarities; and (2) CFGSCDSA-noCGPL, where the confidence-guided pseudo-labeling strategy was excluded from the graph structure learning process. Fig 3D illustrates the ablation results in terms of AUC, AUPR, F1, and recall. CFGSCDSA- noCGPL achieves the lowest scores across all evaluation metrics, indicating that confidence-guided pseudo-labeling strategy plays a crucial role in stabilizing self-supervised learning and enhancing representation quality. Likewise, the performance drop observed in CFGSCDSA-noCFE indicates that the collaborative feature-sharing module is essential for facilitating multi-source feature interactions. In addition, removing both modules results in a substantial reduction in recall, implying that the full CFGSCDSA framework is more effective at identifying true circRNA–drug sensitivity associations. The cooperative effect of confidence-guided pseudo-labeling strategy and collaborative feature learning helps minimize the omission of positive samples.

### 3.5. Case study

To assess the practical significance of CFGSCDSA, we performed case studies on crizotinib and etoposide. Data retrieved from the GDSC database were used to train CFGSCDSA and generate prediction outputs. For each drug, the 20 top-scoring circRNAs candidates were identified and validated through Cancer Therapeutics Response Portal (CTRP) to confirm their reliability. Specifically, circRNA–drug associations annotated as “CTRP” indicate that they are supported by the CTRP database with FDR less than 0.05, whereas entries labeled as “No evidence” denote that no supporting evidence was identified in CTRP. The detailed results are provided in Tables 2 and 3.

**Table 2:** The top 20 circRNAs associated with drug crizotinib.

Ranking	circRNA	Evidence	Ranking	circRNA	Evidence
1	VIM	No evidence	11	EHBP1L1	CTRP
2	POLR2A	CTRP	12	ADPGK	CTRP
3	SPINT2	CTRP	13	SFPQ	CTRP
4	THBS1	CTRP	14	DBN1	No evidence
5	ENO2	No evidence	15	ANKRD36	No evidence
6	ANP32B	CTRP	16	MEF2D	CTRP
7	TCOF1	No evidence	17	CRIM1	CTRP
8	CTTN	CTRP	18	COL1A1	CTRP
9	AATF	CTRP	19	ACTB	No evidence
10	KRT19	CTRP	20	SWAP70	CTRP

**Table 3:** The top 20 circRNAs associated with drug etoposide.

Ranking	circRNA	Evidence	Ranking	circRNA	Evidence
1	VIM	No evidence	11	MEF2D	CTRP
2	POLR2A	CTRP	12	ENO2	No evidence
3	SPINT2	CTRP	13	PTMS	CTRP
4	THBS1	CTRP	14	MUC16	CTRP
5	KRT19	CTRP	15	PEA15	CTRP
6	CTTN	CTRP	16	EFEMP1	CTRP
7	CRIM1	CTRP	17	ESRP2	CTRP
8	ASPH	CTRP	18	FBLN1	CTRP
9	ANP32B	CTRP	19	DBN1	No evidence
10	COL1A1	CTRP	20	ANKRD36	No evidence

Crizotinib, as a tyrosine kinase inhibitor, suppresses the activity of multiple oncogenic receptors, including hepatocyte growth factor receptor, anaplastic lymphoma kinase (ALK), and ROS1 [47]. Based on the CFGSCDSA predictions, 20 circRNAs were identified as potential candidates associated with crizotinib, and notably, 16 of them were validated by CTRP. Several of the top predicted circRNAs, such as those derived from VIM, SPINT2, THBS1, and CTTN, are hosted by genes involved in cytoskeletal remodeling, epithelial–mesenchymal transition, and cell adhesion [48–51]. These processes are known to modulate the activity of receptor tyrosine kinase pathways targeted by crizotinib, providing a biologically plausible link between the predicted circRNAs and drug sensitivity.

Etoposide (VP-16) is widely employed as an antineoplastic agent due to its ability to inhibit DNA topoisomerase II [52,53]. Previous studies have demonstrated that this drug is associated with the development of leukemia. As shown in Table 3, among the top 20 circRNAs potentially associated with etoposide predicted by CFGSCDSA, 17 were validated in the CTRP database. Many of the highly ranked circRNAs for etoposide, including those derived from POLR2A, MEF2D, KRT19, and ASPH, originate from genes associated with DNA repair, transcriptional regulation, and apoptosis [54–57]. Because etoposide induces cytotoxicity through DNA double-strand break accumulation, circRNAs from these host genes may influence cellular responses to DNA damage, thereby affecting drug sensitivity.

In order to assess the performance of CFGSCDSA on novel drug prediction, we selected belinostat and vorinostat and all prior associations were removed. The top 10 predicted circRNAs for each drug are listed in Table 4. Validation results from CTRP showed that seven circRNAs for belinostat and seven circRNAs for vorinostat were successfully confirmed. For the HDAC inhibitors belinostat and vorinostat, several top circRNAs are hosted by genes involved in chromatin remodeling and epigenetic regulation, such as LMNA, JUP, FN1, and CALD1 [58–61]. Since HDAC inhibitors modulate transcriptional activity and chromatin accessibility, circRNAs originating from these pathways may plausibly alter cellular responses to epigenetic therapy.

**Table 4:** The top 10 circRNAs associated with drug belinostat and vorinostat.

Ranking	circRNA	Evidence	Ranking	circRNA	Evidence
1	SPINT2	No evidence	1	ANP32B	CTRP
2	COL1A1	CTRP	2	CALD1	CTRP
3	LMNA	CTRP	3	VIM	CTRP
4	DCBLD2	CTRP	4	ANKRD36	No evidence
5	COL7A1	CTRP	5	JUP	CTRP
6	VIM	CTRP	6	FN1	CTRP
7	ESRP2	No evidence	7	ANKRD36C	No evidence
8	CALD1	CTRP	8	CTSB	CTRP
9	MATN2	CTRP	9	ESRP2	No evidence
10	FN1	CTRP	10	EVPL	CTRP

## 4. Conclusions

This study introduces CFGSCDSA, a model that integrates collaborative learning and self-supervised learning for predicting CDSA. Specifically, CFGSCDSA employs collaborative learning to integrate heterogeneous features, followed by graph structure learning based on confidence-guided pseudo-labeling strategy to refine feature representations. Among seven state-of-the-art methods, CFGSCDSA demonstrates superior performance according to experimental results, and case studies further validate its practical utility. Nevertheless, the model still faces challenges such as data sparsity and class imbalance. Future work will focus on optimizing graph structure learning and addressing imbalance issues. Overall, CFGSCDSA provides a practical valuable framework for CDSA prediction and offers reliable technical support for clinical drug discovery.
